# On Logical and Scientific Strength

**DOI:** 10.1007/s10670-024-00835-2

**Published:** 2024-08-13

**Authors:** Luca Incurvati, Carlo Nicolai

**Affiliations:** 1 Department of Philosophy and Institute for Logic, Language and Computation, University of Amsterdam, Postbus 94201, 1090 GE Amsterdam, The Netherlands; 2 Department of Philosophy, King’s College London, Strand Campus, London, WC2R 2LS UK

## Abstract

The notion of strength has featured prominently in recent debates about abductivism in the epistemology of logic. Following Timothy Williamson and Gillian Russell, we distinguish between logical and scientific strength and discuss the limits of the characterizations they employ. We then suggest understanding logical strength in terms of interpretability strength and scientific strength as a special case of logical strength. We present applications of the resulting notions to comparisons between logics in the traditional sense and mathematical theories.

## Introduction

Scientific theories are standardly thought to be selected on the basis of adequacy to the data and how well they fare with respect to a number of theoretical virtues (van Fraassen, 1980; Lipton, 2004; Keas, 2017). One such virtue is strength, which has been discussed in the philosophy of science. This paper provides an account of the notions of logical and scientific strength. Our focus will be on logical and mathematical theories. However, our account promises to be applicable also in more general contexts, such as scientific theories.

Our study is prompted by the recent interest in logical abductivism. This is the view that logical theories should be selected in the same way as scientific theories. Logical abductivism was famously advocated by Quine (1951), Goodman (1955), and Putnam (1968). It has received much attention in the recent literature as a way to navigate the wide array of non-classical solutions to the logical, set-theoretic and semantic paradoxes (Priest, 2005, 2016; Williamson, 2013, 2017). Logical abductivism promises to provide a way of resolving in a principled manner disputes between rival logics which would otherwise appear hard to settle. Abductivism, so the story goes, replaces clashes of intuition with appeal to criteria for theory choice that are accepted by the broader scientific community. For instance, rather than debating the status of paradoxical sentences, one would determine which semantical theory scores better with respect to those criteria.

According to the logical abductivist, then, theory choice in logic is no different from theory choice in the natural sciences. But the recent revival of interest in abductivism has been associated with the idea that logic is similar to the natural sciences in other respects. This is known as *anti-exceptionalism* about logic. The anti-exceptionalist may hold, for instance, that logical principles are not analytic or (metaphysically) necessary or a priori (Hjortland, 2017). As Russell (2018) and Read (2018) have pointed out, however, some form of exceptionalism is compatible with abductivism. Although our focus is on abductivism, our discussion is clearly relevant for any anti-exceptionalist position which embraces an abductive methodology.

Abductive methodology has been employed also for theory choice in mathematics. Russell (1973) advocated the adoption of the regressive method to justify mathematical axioms. An abductivist-friendly account is famously given by Gödel (1947), who suggested that set-theoretic axioms may be extrinsically justified. More recently, Priest (2006) defended naïve set theory against iterative set theory on the grounds of alleged greater simplicity. A thorough-going abductivist approach to the philosophy of set theory has been advanced by Quine (1990: 95), who argues that considerations of simplicity, economy and naturalness sanction the Axiom of Constructibility. Against this, Maddy (1997) uses the maxims Unify and Maximize to instead reject Constructibility as a candidate axiom for a foundation of mathematics.

In the philosophy of science, van Fraassen (1980: 67–68) distinguishes between *logical* and *empirical* strength. A similar distinction is made by Williamson (2017) and Russell (2018) under the labels of *logical* and *scientific* strength. Roughly speaking, the notion of logical strength of a theory takes into account only its deductive power, whereas the notion of scientific strength has mostly to do with its informational content.

There has been some controversy about the status of the criterion of strength in the recent abductivist literature. Williamson thinks that logical and scientific strength are both virtues and that the former entails the latter. Russell accepts that scientific strength is a virtue but criticizes the view that logical strength should be regarded as one. A more radical position, adumbrated by Hjortland (2017), holds that logical weakness—and therefore the capability of a logic of drawing more distinctions—is a virtue in a theory.

We examine Russell’s and Williamson’s accounts of logical and scientific strength and find them wanting. We suggest understanding logical strength in terms of interpretability strength and scientific strength as a special case of logical strength. The emerging picture contrasts with Russell’s analysis in that it is compatible with considering logical and scientific strength as theoretical virtues, and with Williamson’s in that scientific strength is a special case of logical strength.

## Logical Strength

The aim of this section is to offer a novel account of logical strength. To clear the ground for our account, we first rebut arguments against the status of logical strength as a theoretical virtue and identify problems with extant accounts of logical strength.

### Logical Strength as a Theoretical Virtue

Williamson characterizes logical strength in terms of deductive power. On his account, a theory *T* is *logically stronger* than a theory ${T}^*$ just in case every theorem of ${T}^*$ is a theorem of *T* but not vice versa. This can be extended to consequence relations by saying that a consequence relation $\vdash$ is stronger than a consequence relation $\vdash ^*$ just in case whenever $\vdash ^*$ holds so does $\vdash$ but not vice versa.

Williamson’s characterization of logical strength makes it sound as if the comparison of logical theories is a metalinguistic affair (Williamson, 2017, p. 332). However, Williamson aims to vindicate the idea that it is not. To this end, Williamson suggests comparing logics by encoding their consequence relation via an operator which takes a set of premises as argument and returns the set of its consequences. Thus, if $\Gamma$ is a set of sentences, $\textsf{Cn}(\Gamma )$ is $\{\varphi \;|\; \Gamma \vdash \varphi \}$.^1^ Comparison of logical theories then proceeds by comparing the different $\textsf{Cn}(\Gamma )$s to which the logical theories give rise for different choices of well-confirmed $\Gamma$. However, it should be noted that this strategy still appears to make theory comparison a metalinguistic affair, *contra* Williamson’s intentions. For the set $\textsf{Cn}(\Gamma )$ is individuated via the relation $\Gamma \vdash \varphi$, which is metalinguistic: the elements of $\Gamma$ and $\varphi$ are mentioned rather than used.

Williamson claims that logical strength is a theoretical virtue and that this, together with the fact that simplicity too is a virtue, amounts to a *prima facie* case for classical logic:

Once we assess logics abductively, it is obvious that classical logic has a head start on its rivals, none of which can match its combination of simplicity and strength. Its strength is particularly clear in propositional logic, since PC is Post-complete, in the sense that the only consequence relation properly extending the classical one is trivial (everything follows from anything).

Recently, Gillian Russell (2018) has challenged Williamson’s claims. She agrees with Williamson’s characterization of logical strength but argues that logical strength is neither a theoretical virtue nor a theoretical vice. According to her, if logical strength were a virtue, then, *ceteris paribus*, if theory *T* is logically stronger than theory *S*, *T* is better than *S*. Similarly, if logical strength were a vice, then, *ceteris paribus*, if theory *T* is logically stronger than theory *S*, *T* is worse than *S*. But, she continues, is plainly not the case that, *ceteris paribus*, a theory is always better off (worse off) by having more (less) of logical strength: a theory can have too much or too little logical strength. $\textbf{Triv}$, the trivial logic in which any sentence follows from any set of premisses, is too strong: *snow is white* just does not entail *grass is purple*. $\textbf{Ni}$, the empty logic in which nothing follows from any set of premisses, is too weak: *snow is white and grass is green* do entail *snow is white*.

This argument will not persuade the defender of logical strength as a theoretical virtue. She can happily grant that if a theory is logically stronger than another theory then, all things being equal, it is better; but she will insist that in the case considered by Russell things are not equal. In particular, $\textbf{Triv}$ is plainly not adequate to the data: by entailing everything, the theory sanctions entailments which contradict our intuitions about, say, *grass is green* not following from *snow is white*. Thus, this is just a case, where logical strength is trumped by the fact that the theory is not adequate to the data. As Williamson (2017: 335) puts it: ‘First comes fit with the evidence’. A similar response is available to the defender of logical strength as a *vice*: by entailing nothing, $\textbf{Ni}$ fails to be adequate to the data.

It may be objected that this response does not leave any role to play for strength in theory selection. In particular, it might look as though differences in logical strength between theories are always underwritten by differences in fit with the data. Logics that are too strong or too weak may be ruled out on the basis of their inadequacy to data, without appealing to logical strength in their abductive analysis. The objection may be resisted. First, data may underdetermine a choice of logic and strength can play a role in such cases. A conception of adequacy to the data may require a logic to be non-empty and non-trivial (Williamson, 2017, p. 335), without settling all facts concerning inference patters involving negation. This is compatible with preferring classical logic over paraconsistent or intuitionistic logic on the basis of logical strength, as Williamson does. Second, in standard conceptions of abduction data may be revisable on the basis of other criteria, and there is no reason to rule out strength as one of these.

Similar considerations apply to Read’s response to Williamson abductivist argument for classical logic. Read begins by observing that classical logic is not the only logic to be Post-complete, as witnessed by the case of Abelian logic. He then writes:

A good argument would still ask which logic was the right one: information is not everything, if some of that information is wrong. In the case of Abelian logic, some is indeed wrong: e.g.

** $$\begin{aligned} ((p \rightarrow q) \rightarrow q) \rightarrow p \end{aligned}$$

is valid in Abelian logic, but is simply false (as an account of conditionals).

But we take it that Williamson would agree with much of this: logical strength is not everything and the case for classical logic is to be understood with the proviso that the logic we want ought to also be data adequate. And classical logic’s fit with the evidence can and has been challenged, e.g. by relevant logicians such as Read. One may consider logical strength a virtue whilst taking fit with evidence as another criterion for theory choice.

Indeed, considering logical strength as a virtue is compatible with thinking that this virtue is always trumped by adequacy to the data. In the mathematical context, Maddy comes close to claiming as much. She is arguing in favour of the maxim Maximize, which tells us that we should strive for set theories which are as generous as possible. Maddy is very clear that subscribing to Maximize as a maxim in no way commits one to choosing the most generous of theories—the trivial theory. For, she says, this maxim can be trumped or at least curtailed by other maxims. In particular, she says, ‘consistency is an overriding maxim’ (Maddy, 1997, p. 216).

Thus, extant arguments purporting to refute the idea that logical strength is a virtue fail. Even so, there are a number of issues with Williamson’s characterization of logical strength as inclusion between sets of consequences. First, Williamson’s characterization is not immediately applicable to cases in which one deals with different languages. In general, on Williamson’s characterization, all we can say about the relative strength of two logics featuring disjoint sets of logical constants—such as intuitionistic propositional logic and $\textbf{S4}$—is that they are incomparable. This makes Williamson’s characterization inadequate to select logics using an abductive methodology. On the other hand, our proposal will take into account translations between languages. This will enable us to provide a framework for carrying out abductive comparisons among virtually any logics, including the relation between intuitionistic propositional logic and $\textbf{S4}$.

Another issue with Williamson’s characterization of logical strength concerns its use of the notion of a well-confirmed sentence. The idea is that we can assess a logic by considering $\textsf{Cn}(\Gamma )$ where $\Gamma$ is a set of well-confirmed sentences, such as well-established principles of physics. However, in typical cases, whether the members of $\Gamma$ are well-confirmed or not depends on the background logic of the relevant theory. For instance, whether certain principles of physics can be taken to be well-confirmed depends on whether their consequences fit with the data. But what these consequences are, in turn, may depend on the background logic. Thus, it is not clear that we can find adequate $\Gamma$s which we can take to be well-confirmed independently of the background logic.

Finally, Williamson claims that logical strength entails a ‘looser notion’ of scientific strength, but he does not provide a detailed account of scientific strength and of why such an entailment should obtain. In fact, in what follows we will provide a detailed account of scientific strength and of its relationship with logical strength in which such an entailment will fail.

We will offer a characterization of logical strength that overcomes the issues faced by the notions employed by Williamson and Russell. The notion of logical strength we propose is based on the notion of translation and applies to theories formalized in different signatures. As such, it will be more encompassing than Williamson and Russell’s notions while remaining faithful to the idea that logical strength has to do with deductive strength. Moreover, our characterization will extend more naturally so as to apply beyond the purely logical part of a theory. Finally, our characterization will form the basis of a detailed account of scientific strength.

However, it should be stressed that, in providing a characterization of logical strength and scientific strength, we are not taking a stance on whether these features should ultimately be considered as virtues, vices or neither. Instead, our aim is to provide a framework for comparing the strength of theories which can be used in debates over whether strength is a virtue, a vice, or neither. For instance, in a view similar to Maddy’s above, logically stronger theories should, all things being equal, be preferred in view of their being more “generous”. The notion of strength in question cannot be understood in terms of deductive strength, as one would like to compare mutually inconsistent theories in the same language.^2^ The notion of logical strength we propose can be employed in such cases to make sense of this view of logical strength as a virtue.

### Characterizing Logical Strength

In our view, the strength of a theory has to do solely with the structure of its derivations. A theory is as strong as another if the former can mimic the inferential structure of the latter. As mentioned, translations allow us to compare theories in different languages. We propose to compare the strength of theories in terms of the existence of suitable translations between them. In order to be suitable, translations should be uniform procedures allowing one to recover the structure of derivations of a theory.

In comparing theories with respect with their logical strength, we allow logical and non-logical primitives to be reinterpreted as long as the basic structure of derivations is adequately recovered. Any other notion of strength which demands preservation of information in the logical or non-logical component of theories would not count as *logical* strength, because it would not abstract away as much as possible from specific content. For instance, our notion of logical strength is not sensitive to semantic properties of theories, such as soundness. Prototypical examples of unsound theories are obtained by extending a theory *T*, which is presumed to be sound, with a canonical inconsistency statement $\lnot \textrm{Con}(T)$ stating that there is a formal proof of a contradiction in *T*. Accordingly, our characterization entails that Peano Arithmetic ($\textsf{PA}$) and $\textsf{PA}+\lnot \textrm{Con}(\textsf{PA})$ have equal logical strength. This is essentially because $\lnot \textrm{Con}(\textsf{PA})$ can be translated in $\textsf{PA}$ in a way that preserves its role in derivations while re-interpreting the notion of provability involved in the consistency statement.^3^ As we will see later on, what distinguishes logical and scientific strength of theories is how specific information contained in their logical or non-logical primitives is handled. In particular, in comparing the scientific strength of theories we will impose stricter conditions on how specific information contained in the primitive concepts of theories is preserved under suitable translations.

We now implement these ideas into a formal framework for logical strength. For our technical development, it’s useful to view logics as sets of inferences closed under specific rules. Mathematical theories are then special cases of such inferences where the premiss set is a fixed set of axioms. When comparing mathematical theories in *classical logic*, it is customary to say that a translation from a language $\mathscr {L}_1$ to a language $\mathscr {L}_2$ consists of an ordered pair $\tau = \langle \delta , F \rangle$ where $\delta$ is the domain of the translation and *F* is a recursive mapping associating each *n*-ary relation symbol $R(y_1,\ldots ,y_n)$ of $\mathscr {L}_1$ with an $\mathscr {L}_2$-formula $F(R)(y_1,\ldots ,y_n)$. The translation $\tau$ commutes with the connectives and $\delta$ relativizes the quantifiers so that, e.g. $(\forall x\varphi )^\tau := \forall x(\delta (x)\rightarrow \varphi ^\tau )$. An interpretation is then a translation that preserves provability. Specifically, a translation $\tau$ from the language $\mathscr {L}_1$ of a theory ${T}_1$ to the language $\mathscr {L}_2$ of a theory ${T}_2$ is an interpretation of ${T}_1$ into ${T}_2$ if for every set of $\mathscr {L}_1$-sentences $\Gamma$ and $\mathscr {L}_1$-sentence $\varphi$, if $\Gamma \vdash _{{T}_1} \varphi$, then $\Gamma ^\tau \vdash _{{T}_2} \varphi ^\tau$ (where, as usual, $\Gamma \vdash _{T} \varphi$ is a shorthand for $\Gamma \cup T \vdash \varphi$, and $\Gamma ^\tau$ is $\{\varphi ^\tau | \varphi \in \Gamma \}$).^4^

Finally, ${T_1}$ and ${T_2}$ are mutually interpretable if ${T_1}$ is interpretable in ${T_2}$ and vice versa. Given these definitions, we could then characterize logical strength for theories in classical logic by saying that a theory ${T_1}$ has greater or equal logical strength than a theory ${T_2}$ just in case there is an interpretation of ${T_1}$ in ${T_2}$, and that they have the same logical strength just in case they are mutually interpretable. However, since we aim to deal with mathematical theories formulated in a given non-classical logic as well, we generalize the notion of interpretation above, and call a translation from $\mathscr {L}_1$ to $\mathscr {L}_2$ any recursive mapping that associates formulas of $\mathscr {L}_2$ with primitive concepts of $\mathscr {L}_1$ and that is recursively extended to more complex formulas by suitably commuting with the logical constants. An interpretation is then a translation that preserves provability in a such given logic.

Let us consider a few examples that will be relevant also for our later discussion. As mentioned, unsound extensions of sound theories obtained via inconsistency claims have equal logical strength, for instance $\textsf{PA} +\lnot \textrm{Con}(\textsf{PA})$ and $\textsf{PA}$. But there are, of course, also pairs of not obviously unsound theories that have equal logical strength, such as $\textsf{ZFC}$ (Zermelo-Fraenkel set theory with the Axiom of Choice) and $\textsf{ZF}$. To consider theories formulated in different languages, theories of finite mathematics such as the arithmetical $\textsf{PA}$ and the set-theoretic $\mathsf {ZF_{Fin}}$ ($\textsf{ZF}$ with the Axiom of Infinity replaced by its negation) also have equal logical strength.^5^ To mention examples of non-classical theories, the theory $\textsf{PA}_\textsf{k3}(P)$—that is Peano arithmetic formulated in the three-valued Strong-Kleene logic $\textbf{K3}$ and in the language with an additional predicate *P*, whose interpretation may not be classical—is mutually interpretable (relative to the logic $\textbf{K3}$) with $\textsf{PA}_\textsf{k3}(P)$+$\lnot \textrm{Con}(\textsf{PA}(P))$.^6^ Hence, the proposed characterization entails that the two theories have the same logical strength.

The characterization of logical strength in terms of mutual interpretability provides a precise formal counterpart to the idea that logical strength resides in a theory’s capability of mimicking inferential structures, possibly via translations that reinterpret primitive concepts. However, the characterization is not sufficient to deal with all cases of comparison of logical strength. For instance, we want to be able to compare pure logics, and in that case we want to reinterpret the logical vocabulary itself, whereas the standard notion of interpretation is designed so as to leave the logical vocabulary alone.

Whilst we cannot hope to preserve the meanings of the connectives when translating between logics, it seems that a translation between logics, besides the basic requirement of being recursive, ought at least to (i) be uniform so that, e.g., it is not the case that $p \wedge q$ is translated as $p \vee q$ but $r \wedge s$ is translated as $r \rightarrow s$ and (ii) allow going beyond translating each operator with a single operator, e.g. we want to be able to translate, say, $p \wedge q$ as $\lnot (\lnot p \vee \lnot q)$.^7^; finally, for our purposes it’s important that (iii) a suitable translation is sensitive to triviality. In other words, we require that an absurdity is preserved under the translation: for instance, the classical absurdity $P\wedge \lnot P$ cannot be translated into a tautology, say $\top \wedge P$, in Strong Kleene logic $\textbf{K3}$, although this translation would satisfy the previous requirements. A suitable notion of translation is the notion of a *schematic translation* (Prawitz & Malmnäs, 1968; Wojcicki, 1988; Pellettier & Urquhart, 2003). The general idea is that a translation is schematic if the translation of a complex formula is a fixed schema of the translation of its parts. As a result, formulae instantiating the same schema are translated in the same way. So, for instance, if $p \wedge q$ is translated as $p \vee q$, then $r \wedge s$ must be translated as $r \vee s$. But it *is* possible to translate $p \vee q$ as $\lnot (\lnot p \wedge \lnot q)$. To address (iii), from now on we only consider schematic translations that preserve absurdity. Although the notion of absurdity preservation is logic-relative, and as such we cannot provide a general definition of this requirement, for each specific case study considered below we can provide a precise definition of this notion. Moreover, for the specific translations considered below, a fully general method to enforce preservation of absurdity is to require admissible translations to be *compositional* (French, 2010, p. 16).^8^

To define the notion of a schematic translation, we first define the notion of a *schema*. A schema is a map from formulae (and possibly variables) to the formulae instantiating a *schema-string*, i.e. an expression featuring metalinguistic variables such as $\varphi \vee \psi$ or $\forall \alpha \varphi$. We say that a translation from the language $\mathscr {L}_1$ of a logic $L_1$ to the language $\mathscr {L}_2$ of a logic $L_2$ is *schematic* if it is a recursive mapping $\tau$ such that (i) each atom *p* of $\mathscr {L}_1$ is assigned a $\mathscr {L}_2$ formula, and (ii) for each piece $\spadesuit$ of logical vocabulary in $\mathscr {L}_1$ there is an $\mathscr {L}_2$-schema $\mathscr {T}$ such that for all sequences $\varphi _1, \ldots , \varphi _\gamma$ of $\mathscr {L}_1$-formulae $(\spadesuit \varphi _1, \ldots , \varphi _\gamma )^\tau := \mathscr {T}(\varphi _1^\tau , \dots , \varphi _\gamma ^\tau )$. A schematic translation $\tau$ from $\mathscr {L}_1$ to $\mathscr {L}_2$ is *sound* if it preserves provability, that is if for every $\Gamma$ and $\varphi$ in the language of ${L}_1$, we have that if $\Gamma \vdash _{L_1} \varphi$ then $\Gamma ^\tau \vdash _{L_2} \varphi ^\tau$. A schematic translation $\tau$ from $\mathscr {L}_1$ to $\mathscr {L}_2$ is *exact* if it also preserves unprovability, namely if for every $\Gamma$ and $\varphi$ in the language of ${L}_1$, we have that $\Gamma \vdash _{L_1} \varphi$ if and only if $\Gamma ^\tau \vdash _{L_2} \varphi ^\tau$.

Schematic translations played a prominent role in the history of logic. Gödel, via the so-called *negative translation*, showed that there is a (exact) schematic translation of classical logic into intuitionistic logic. In doing so, he established the consistency of classical logic and classical arithmetic (Peano Arithmetic) relative to their intuitionistic counterparts. He also provided the basis of provability logic, justification logic, and Kripke semantics for intuitionistic logic by providing a schematic translation of the latter logic into the modal logic $\textbf{S4}$.

We take sound schematic translatability to be a core component of our account of logical strength. In fact, if we were dealing just with logics, we could simply characterize logical strength by saying that a logic $L_1$ has greater or equal logical strength than a logic ${L_2}$ just in case there is a sound schematic translation of $L_2$ in ${L_1}$, and that they have the same logical strength if this holds mutually. One would obtain a different notion of logical strength with exact translations instead of sound ones. Although we believe this to be an alternative worth exploring, we here focus on sound schematic translations in order to preserve the intuitive idea that $L_1$ being a sublogic of $L_2$ implies that $L_2$ is at least as (logically) strong as $L_1$. Once again, we emphasize that the sound schematic translations we consider preserve absurdity, and so we are in fact considering a specific subclass of sound schematic translations.

So far we have only afforded the means of comparing either different logics or mathematical theories cast in the same background logic. However, we also want to be able to compare mathematical theories cast in different logics. For instance, we want to compare the logical strength of $\textsf{ZF}$ and Heyting Arithmetic ($\textsf{HA}$), the theory whose axioms are those of $\textsf{PA}$ but whose logic is intuitionistic logic rather than classical logic.

Cases of this sort lead us to our full characterization of the notion of logical strength, which is obtained via a two-stage process and subsumes the characterizations of logical strength that would be suitable in the case of logics or in the case of theories cast in the same logic. Given a theory ${T_1}$ with logic ${L_1}$ and a theory ${T_2}$ with logic ${L_2}$, the idea is that to determine whether ${T_1}$ is at least as strong as ${T_2}$ one first schematically interprets ${L_2}$ into ${L_1}$ and then interprets ${T_2}$ (under the logic ${L_1}$) into ${T_1}$.

Logical strength $T_1$ is *at least as logically strong as* $T_2$ iff there is a sound schematic translation $\tau$ of the logic $L_2$ of $T_2$ in the logic $L_1$ of $T_1$, and there is an interpretation (relative to the logic $L_1$) of $T^\tau _2$ in $T_1$.

We say that $T_1$ is *logically stronger* than $T_2$ if $T_1$ is at least as logically strong as $T_2$ but not *vice versa*. Our definitions entail that, for mathematical theories formulated in classical logic, logical strength coincides with the familiar notion of interpretability strength. More generally, for mathematical theories in a given logic, logical strength coincides with the notion of interpretability strength relative to that logic. Similarly, when comparing purely logical systems, our characterization of logical strength reduces to the existence of a sound schematic translation, since we are taking logics to be theories with the empty set of non-logical principles.

We now discuss some applications of our characterization. We begin by considering cases of comparison between logics. A simple example involves variations of (classical) propositional logics.^9^ Propositional logic formulated with logical constants $\{\lnot ,\vee \}$ can be translated exactly into the pure implicational fragment of classical propositional logic with a distinguished sentence letter standing for falsum, so the latter is at least as logically strong as the former. Since schematic interpretability preserves undecidability, it is clear that classical predicate logic is logically stronger than classical propositional logic.^10^ The Gödel–Gentzen translation (Troelstra & Schwichtenberg, 2003, Sect. 2.3) is an exact schematic translation of classical logic into intuitionistic logic. Therefore, intuitionist logic can mimic the structure of classical derivations—modulo reinterpreting some logical vocabulary. As a consequence, intuitionistic logic is as strong as classical logic. Moreover, intuitionistic logic is a sublogic of classical logic, and hence it can be trivially (schematically) translated in a sound way into into classical logic. Hence, intuitionistic logic and classical logic have equal logical strength. The Gödel–McKinsey–Tarski translation is an exact schematic translation of intuitionistic logic into $\textbf{S4}$. Hence, $\textbf{S4}$ is at least as logically strong as intuitionistic logic. Similarly to the previous case, we can also reproduce the structure of $\textbf{S4}$-derivations into intuitionistic logic.^11^ Thus, $\textbf{S4}$ and intuitionistic logic have the same logical strength. In the context of comparison between modal logics, by translating □$A$ with □$A\wedge A$, one can show that the modal logics $\textbf{K}$ and $\textbf{T}$ have the same logical strength.

We now turn to applications of our notions to non-logical axioms. The full power of our characterization of logical strength comes into play when we consider theories formulated in different logics. For instance, our notion enables us compare $\textsf{ZF}$ to $\textsf{HA}$. It is well-known that $\textsf{ZF}$ has greater interpretability strength than $\textsf{PA}$—and therefore, according to our characterization, greater logical strength. On our picture, the same remains true if arithmetic is formulated in intuitionistic rather than classical logic. The details are as follows. Clearly, there is a sound translation of intuitionistic logic into classical logic such that:

$$\begin{aligned} \textsf{HA} \vdash _{\textsf{IL}} \varphi \Rightarrow \mathsf{HA^{id}} \vdash _\textsf{CL} \varphi ^\textsf{id}. \end{aligned}$$

Then one simply interprets $\mathsf{HA^{id}}$ formulated in classical logic—that is, $\textsf{PA}$—in $\textsf{ZF}$ by means of the interpretation that relativizes quantification over natural numbers as quantification over finite ordinals. Since there is no interpretation of $\textsf{PA}$ in $\textsf{ZF}$, this establishes that $\textsf{ZF}$ is logically stronger than $\textsf{HA}$. A similar phenomenon holds true when one considers set theories in intuitionistic logic and compares them with classical arithmetic: intuitionistic $\textsf{ZF}$ ($\textsf{IZF}$ for short) is logically stronger than $\textsf{PA}$.^12^ To see this, one first employs the Gödel–Gentzen translation $\textsf{gg}$ to obtain:

$$\begin{aligned}\textsf{PA} \vdash _{\textsf{CL}} \varphi \Rightarrow \textsf{PA}^\textsf{gg} \vdash _\textsf{IL} \varphi ^\textsf{gg}\end{aligned}$$

Then one would need to show that $\textsf{PA}^\textsf{gg}$, qua subtheory of $\textsf{HA}$, is interpretable in $\textsf{IZF}$.^13^ Since $\textsf{IZF}$ has (much) higher consistency strength than $\textsf{HA}$, there is no interpretation of the former in the latter theory.

The examples just discussed lend support to the adequacy of our characterization of logical strength based on preservation of inferential structure. One of the main advantages of our characterization is its generality. We are able to compare both logics and theories, and various combination thereof. We believe this generality is essential to the abductive comparison of logics and theories. Without the possibility of comparing theories with different logical and non-logical primitive vocabulary, there is little hope for logical abductivism to succeed.

Yet another advantage of our characterization is that it leads naturally to a precise characterization of scientific strength. It is to this issue that we now turn.

## Scientific Strength

In this section we first discuss Williamson’s and Russell’s accounts of scientific strength. We then propose our own account.

### Williamson and Russell on Scientific Strength

Williamson (2017) holds that logical strength entails a ‘looser’ notion of *scientific strength*. For instance, since classical logic proves all instances of $\varphi \vee \lnot \varphi$ and intuitionistic logic doesn’t, the former is logically stronger, but also scientifically stronger than the latter: according to Williamson, a general claim—all instances of excluded middle are valid—is scientifically more informative than its negation. Similarly, ‘the time between 3:14 and 3:16’ is more informative than ‘the time between 4:00 and 12:00’. So, although Williamson does not provide a detailed account of scientific strength, both logical form and a certain degree of accuracy are relevant for his view.

Russell (2018) rejects Williamson’s claim that logical strength implies scientific strength. She does so by distinguishing between two senses of scientific strength. According to the first, a logic *L* is scientifically strong if it is able to decide, for each argument form in a given language, whether the argument is *L*-valid or not. In this first sense, each logic is as strong as another, no matter how different they are in logical strength: each logic partitions the set of all argument forms into valid and invalid.

Russell describes her second sense of scientific strength as follows:

If our question is ‘which instances of LL can we use?’ (where LL is some disputed logical law) then the logically stronger logic tells us ‘all of them’ whereas the weaker logic says ‘not all of them’—and this tells us nothing further about which particular instances are untarnished (Russell, 2018, p. 12).

In this second sense the trivial logic **Triv** is the strongest logic, because to the question ‘How many instances of the argument form $(\Gamma ,\varphi )$ can we use?’ it answers ‘All of them’. Classical logic would then seem to be scientifically weaker than **Triv**, but stronger than, say, its logically weaker sublogics $\textbf{K3}$, the Logic of Paradox $\textbf{LP}$, and First Degree Entailment $\textbf{FDE}$. There are in fact some argument forms $(\Gamma ,\varphi )$ of which, unlike **Triv**, classical logic can accept only some instances. Similarly, there are familiar argument forms, such as $(\Gamma ,\varphi \vee \lnot \varphi )$ or $(\{\varphi ,\lnot \varphi \},\psi )$, whose instances are uniformly licensed by classical logic but fail to be so in $\textbf{K3}$, $\textbf{LP}$, or $\textbf{FDE}$. Therefore, it would seem that there is a sense of scientific strength that is entailed by logical strength. However, Russell claims that this conclusion would be hasty: any sublogic of **Triv** can be extended to a logic that decides which instances of an argument form are acceptable, and which aren’t. In other words, each logic can be extended in such a way that, to the question ‘How many instances of the argument form $(\Gamma ,\varphi )$ can we use?’, it no longer provides the uninformative answer ‘Not all of them’. Instead, the question is answered by providing a list of acceptable and unacceptable instances. Russell calls this process ‘$\textbf{Triv}$ recapture’. Now any logic that is subject to the procedure of ‘**Triv** recapture’ ends up being as informative as another. Since this equally applies to logic with substantially different logical strength, Russell concludes that there is no sense of scientific strength that is implied by logical strength.

We believe that both accounts of scientific strength offered by Russell have undesirable features. We start with Russell’s first account: on this view, all logics are on a par with respect to scientific strength because either $\Gamma \vDash _L \varphi$ or $\Gamma \nvDash _L \varphi$: according to Russell, a well-defined consequence relation is cast in a set-theoretic (classical) metatheory (Russell, 2018, p. 557). However, it’s clear that under this characterization the specific properties of consequence relations are not relevant at all for their scientific strength. In fact, it is simply a feature of Russell’s classical metatheory that excluded middle holds for logical consequence claims. It follows that, as long as a notion of consequence is well-defined, any logic is as strong as it could be. But if the notion of scientific strength is to play any role in abductive methodology, then it should be capable of discriminating at least between some logics.

To avoid such an essential dependence on classical metatheory, one might try to generalize Russell’s first definition of scientific strength by requiring that each logic *L* is as strong as another one *by its own light*. On this reading, however, Russell’s claims cannot be true in general. There is nothing that guarantees that the notion of logical consequence we are employing satisfies bivalence. For instance, if our metatheory is formulated in a paracomplete setting governed by the logic $\textbf{K3}$, it won’t in general be the case that ‘$\varphi$ follows from $\Gamma$ or it’s not the case that $\varphi$ follows from $\Gamma$’, because the very notion of consequence may be partial (Nicolai & Rossi, 2018). Moreover, in such a scenario, it would seem that logical strength does indeed in many cases entail scientific strength. For instance, classical logic *is* able, for each $\Gamma ,\varphi$, to determine whether $\Gamma \vDash \varphi$ or $\Gamma \nvDash \varphi$, whereas $\textbf{FDE}$ and $\textbf{K3}$ cannot.

It may be objected that non-classical metatheories formulated in a many-valued logic (with finitely many values) still classify arguments according to the relevant truth values. For instance, a meta-theory formulated in $\textbf{K3}$ would classify arguments into valid, invalid, and neither valid nor invalid. Hence, so the objection goes, such a non-classical (meta)logic would have the same scientific strength as any other, including a classical one. However, the reaction would not succeed as it assumes a classical meta-metatheory. If the meta-metatheory is not classical, there is no guarantee that the meta-theory will classify all arguments. For instance, in the case of $\textbf{K3}$, the meta-theory would be silent about the arguments that are neither valid nor invalid. All in all, an entanglement of the debate on theory choice in logic with strong meta-theoretic properties is potentially problematic. It would be desirable to be able to compare logics on the basis of meta-theory so weak that can be shared by the different logics to be compared. In our account, comparison of strength between logics will be realized via the notion of translation, which presupposes only minimal meta-theoretic facts.

Russell’s second sense of scientific strength is based on the notion of **Triv** recapture: any logic *L* can be consistently extended to a logic that decides which instances of a given argument form are valid or not. This understanding of scientific strength faces serious difficulties too. First, it is worth noticing that Russell’s **Triv** recapture is substantially different from standard recapture strategies found in the literature on semantic paradoxes. Let us consider the case-study discussed by Russell. If one’s language amounts to a formal syntax plus a truth predicate $\textrm{Tr}\,$, one can provide models of transparent truth—$\textrm{Tr}\,\ulcorner A\urcorner$ is intersubstitutable with *A* in every context—that satisfy classical logic for all sentences without $\textrm{Tr}\,$. In other words, if $\mathscr {L}_\textrm{Tr}\,:=\mathscr {L}\cup \{\textrm{Tr}\,\}$ is the language under consideration, one can consistently formulate a logic that satisfies all classical principles for $\mathscr {L}$ and the nonclassical principles for $\mathscr {L}_\textrm{Tr}\,$. This is what is often called ‘classical recapture’ (Field, 2008; Beall, 2013).

However, this form of recapture is not sufficient for Russell’s purposes. She requires something much stronger—what she calls Small Square Completeness: for *any* argument form in a given language, one has to be able to decide which instances are licensed and which aren’t. For instance, each specific instance of the form $\textrm{Tr}\,\ulcorner \varphi \urcorner \vee \lnot \textrm{Tr}\,\ulcorner \varphi \urcorner$ must be decided one way or another. This is a hugely complex task. If $\textrm{Tr}\,\ulcorner \varphi \urcorner$ is interpreted via fixed-point semantics in the style of Kripke (1975), the problem at hand reduces to a decision procedure for the set of paradoxical, or ungrounded sentences. Unlike the simple syntactic decision problem underlying recapture strategies, already in the simplest Kripkean setting (the minimal fixed point) this problem is highly non-effective (Burgess, 1986). And these problems become much more complex for more sophisticated constructions such as other Kripkean fixed points, the revision extensions in Gupta and Belnap (1993), the theory of Field (2008), just to mention a few. Moreover, the complexity of the procedure envisaged by Russell is only going to increase if we move from the specific language $\mathscr {L}_\textrm{Tr}\,$ to less rarefied languages closer to English. Therefore, the procedure of $\textbf{Triv}$ recapture is simply unmanageable; it is not the case that any logic can be consistently extended to a Small-Square Complete *logic*, unless by logic we mean extensions of highly non-effective infinitary logics whose set of validities is much more complex than the provable sentences of any recursively axiomatised theory. To be sure, we are not claiming is some specific key cases arguments can be classified as valid or invalid, but only that this is unmanageable for *all* arguments. For instance, a paracomplete theorist may reasonably hold that their theory of truth does not entail the Liar sentence. It’s just that they won’t be able to generalize this to a classification of all arguments into valid or invalid.

### Characterizing Scientific Strength

We now come to our approach to scientific strength. Our proposal shares with Williamson’s the idea that scientific strength is more closely related to the informativeness of a theory than logical strength is. Our proposal goes further in that scientific strength is obtained by placing extra conditions on the relation of being logically stronger. Thus, scientific strength entails logical strength.

Intuitively, logical strength is a coarser grained relation than scientific strength in that it has to do only with the deductive structure of theories, and hence allows for radical re-interpretation of logical and non-logical vocabulary in derivations. Scientific strength is then obtained by supplementing logical strength with stricter conditions so as to preserve information contained in the theories’ primitives. In particular, we no longer allow radical re-interpretations of primitives, but we impose conditions on the preservation, in derivations, of some structural aspects of logical and non-logical constants of theories. For instance, we have seen that $\textsf{PA}$ and $\textsf{PA}+\lnot \textrm{Con}(\textsf{PA})$ have equal logical strength, because the arithmetical primitives used to define provability in $\lnot \textrm{Con}(\textsf{PA})$ can be re-interpreted by $\textsf{PA}$ in a way that does not entail its inconsistency. However, $\textsf{PA}$ and $\textsf{PA}+\lnot \textrm{Con}(\textsf{PA})$ will not have the same scientific strength, because our extra conditions on interpretations will require the role in derivations of $\lnot \textrm{Con}(\textsf{PA})$ to be preserved in a much more accurate way.

We formally render these ideas by means of the notion of intertranslatability. Intertranslatability is also known as definitional equivalence (Glymour, 1970) and synonymy (De Bouvère, 1965; Pellettier & Urquhart, 2003). Earlier we distinguished between interpretations, which relate theories with non-logical axioms in the same logic, and schematic translations, which relate logics. Analogously, we now define intertranslatibility as applied to both cases. The basic idea behind intertranslatablity is to impose extra-conditions on sound schematic translations (in the case of logics) and interpretations (in the case of theories); in addition to requiring sound schematic translations or interpretations that relate two logics or theories, intertranslatability demands the two translations or interpretations to be inverse to each other, provably in these logics or theories.

Logics $L_1$ and $L_2$ are *intertranslatable* if and only if there are sound schematic translations $\sigma$ from the language $\mathscr {L}_{1}$ of $L_1$ to the language $\mathscr {L}_{2}$ of $L_2$ and $\tau$ from $\mathscr {L}_{2}$ to $\mathscr {L}_{1}$ such that^14^

$$\begin{aligned}&\varphi \dashv \vdash _{L_1} (\varphi ^{\sigma })^\tau \text { for any formula }\varphi \text { of }\mathscr {L}_1;\\&(\varphi ^{\tau })^\sigma \dashv \vdash _{L_2} \varphi \text { for any formula }\varphi \text { of }\mathscr {L}_2. \end{aligned}$$

Similarly, one says that theories *S* and *T* in a given logic are intertranslatable if there are interpretations $\sigma$ from *S* to *T*, and $\tau$ from *T* to *S* (with both $\sigma$ and $\tau$ relative to the given logic) such that

$$\begin{aligned}&\varphi \dashv \vdash _{S} (\varphi ^{\sigma })^\tau \text { for any formula }\varphi \text { of }\mathscr {L}_S;\\&(\varphi ^{\tau })^\sigma \dashv \vdash _{T} \varphi \text { for any formula }\varphi \text { of }\mathscr {L}_T. \end{aligned}$$

Since we are dealing both with pure logics and theories featuring non-logical axioms, we again need to characterize scientific strength in terms of a two-step process.

Intuitively, the idea behind our characterization is that a theory *T* (where, recall, logics are limiting cases of theories) is scientifically stronger than another theory *S* if there is some subtheory of *T* that can faithfully reproduce the logical and non-logical information contained in the inferential structure of *S*. The idea of ‘faithfully reproducing’ is captured in the strict requirement imposed to the translation by the notion of intertranslatibility. In particular, intertranslatability requires that both theories recognize (via provability) that the translations that relate them are ‘companion’ to each other in the way they process the original information: when the two translations are suitably combined, they return the original information.

Scientific strength A theory $T_1$ is scientifically as strong as $T_2$ if (i) $T_1$ is at least as logically strong as $T_2$, (ii) the logic $L_2$ of $T_2$ is intertranslatable with a sub-logic of $L_1$ which is either $L_2$ itself or a logically weaker logic—witnessed, say, by $\tau :L_2\rightarrow L_1$—, and (iii) there is a subtheory (sublogic) $T_0$ of $T_1$ which is either $T^\tau _2$ itself or a logically weaker $T_0$ such that $T^\tau _2$ is intertranslatable with $T_0$ (with respect to the logic $L_1$).

Condition (i) in the characterization of scientific strength may be dropped in certain, well behaved cases, for instance when we deal with mathematical theories cast in classical logic. However, we chose to keep it in the general case because we aim to provide a template to deal with a large class of logics, for which the notion of interpretation may be underspecified. This makes it difficult to prove that condition (i) is redundant in full generality.

We now show that the definition delivers intuitively acceptable verdicts on the comparative scientific strength of theories. We start with examples of theories formulated in the same logic. Since scientific strength entails logical strength, it obviously follows that any theories that do not have the same logical strength do not have the same scientific strength either. For instance, $\textsf{ZFC}$ plus the assertion that there exists a inaccessible cardinal is scientifically stronger than $\textsf{ZFC}$ which, in turn, is scientifically stronger than $\textsf{PA}$. For *T* a reasonable classical theory containing a modicum of arithmetic, $T+\textrm{Con}(T)$ is logically stronger than *T*, and properly so, since $T+\textrm{Con}(T)$ is not interpretable in *T* (Lindström, 2003, Ch. 7). It is worth noticing that $\textrm{Con}(T)$ is a $\Pi ^0_1$-sentence of the language of arithmetic, i.e. a purely universal claim. In general, the addition of an independent $\Pi ^0_1$-sentence results in a scientifically stronger theory. This last example obviously extends to theories in different languages that interpret a sufficiently strong arithmetical theory. So our characterization of scientific strength vindicates Williamson’s claim that a universally quantified sentence adds informativeness to a theory. More generally, our characterization entails that a theory is always scientifically as strong as any of its subtheories.

Theories that have the same logical strength may or may not have the same scientific strength. We begin with cases of theories that have the same scientific strength as well as the same logical strength. Some of these theories belong to different mathematical domains, which exhibits one advantage of our definition. Certain set theories with and without urelemente have the same scientific strength. Löwe (2006) shows that $\textsf{ZF}$ and $\textsf{ZF}$ plus countably many urelemente are intertranslatable. A similar phenomenon concerns $\textsf{ZFC}$ and $\textsf{ZFA}$ ($\textsf{ZFC}$ without Foundation plus Aczel’s (1988) Anti-Foundation Axiom). $\textsf{ZFC}$-sets can be interpreted in $\textsf{ZFA}$ as well-founded sets. $\textsf{ZFA}$-sets can be interpreted in $\textsf{ZFC}$ as equivalence classes of graphs with lowest rank. Such interpretations yield the intertranslatability of the two theories (Visser & Friedman, 2014). This example shows that sameness of scientific strength does not amount to sameness of meaning of the theories’ primitives, but only to equivalence with respect to salient aspects of a theory’s primitives. Moving to theories formulated in different signatures, consider, for instance, the theory $\mathsf {ZF_{Fin}}$. Although this theory is not intertranslatable with $\textsf{PA}$ (Enayat et al., 2011, Thm. 5.1), it becomes so once one adds to it the claim that every set has a transitive closure (Kaye & Wong, 2007).

Crucially, our analysis of scientific strength yields natural counterexamples to Williamson’s implication from logical to scientific strength. We now consider cases of theories that have the same logical strength but different scientific strength. A striking example concerns set theory with and without the axiom of choice. In particular, $\textsf{ZFC}$ is not intertranslatable with $\textsf{ZF}$. Therefore, $\textsf{ZFC}$ is scientifically stronger than $\textsf{ZF}$ (Enayat, 2016).^15^ This nicely fits with the intuition that the addition of the axiom of choice to ZF, although innocent from the point of view of mere consistency strength, results in an increase of informativeness of the axioms. Similarly, although adding the Continuum Hypothesis or its negation to $\textsf{ZFC}$ does not increase its logical strength, it does increase its scientific strength. As anticipated, canonical consistency statements display a similar behaviour: although $\textsf{PA}+\lnot \textrm{Con}(\textsf{PA})$ has the same logical strength as $\textsf{PA}$, it is scientifically stronger than $\textsf{PA}$. To establish that $\textsf{PA}+\lnot \textrm{Con}(\mathsf{PA)}$ is as scientifically strong as $\textsf{PA}$ it suffices to notice that $\textsf{PA}$, qua subtheory of $\textsf{PA}+\lnot \textrm{Con}(\mathsf{PA)}$, is trivially intertranslatable with itself. However, $\textsf{PA}+\lnot \textrm{Con}(\mathsf{PA)}$ is neither intertranslatable with $\textsf{PA}$ nor with any logically weaker subtheory of $\textsf{PA}$ (Visser, 2006, Cor. 9.4). A similar phenomenon holds for $\mathsf{ZF(C)}$ and $\mathsf{ZF(C)}+\lnot \textrm{Con}(\mathsf{ZF(C)})$, as well as full second-order arithmetic $\textsf{Z}_2$ and $\textsf{Z}_2+\lnot \textrm{Con}(\mathsf{Z_2})$.^16^

These examples enable us to clarify the dialectic between logical and scientific strength in our setting. Let’s consider the notion of interpretation between theories in classical logic, the key notion involved in determining logical strength. Interpretations can only recover the logical structure of theorems of the interpreted theory, allowing for a radical re-interpretation of its primitives. In our view, this does not suffice to preserve information. Thus, if someone wanted to consider logical strength a virtue, they wouldn’t be able to so on the basis of greater informativeness of the interpreting theory. Nonetheless, they could still do so on account of the interpreting theory recovering the logical structure of theorems. For instance, any proof of inconsistency in the logically weaker theory would entail an inconsistency in the logically stronger theory, but not vice versa. Thus some aspects of the logically weaker theory are preserved by the logically stronger theory and not the other way around. It is not implausible to consider preservation of these aspects a virtue. But, to stress, the aspects of the interpreted theory that are preserved need not be regarded as part of its information.

We now turn to the comparison of logics. We saw in Sect. 2.2 that classical predicate logic is logically stronger than classical propositional logic. Since classical propositional logic is a subtheory of classical predicate logic, it follows that classical predicate logic is also scientifically stronger than classical propositional logic. We can also show that classical propositional logic is scientifically stronger than the many-valued propositional logics $\textbf{K3}$, $\textbf{LP}$ and $\textbf{FDE}$. That classical propositional logic is as scientifically strong as $\textbf{K3}$, $\textbf{LP}$ and $\textbf{FDE}$ obtains because of the sublogic relation. For the other direction, we can show that none of $\textbf{K3}$, $\textbf{LP}$ and $\textbf{FDE}$ can define the classical connectives. Since intertranslatability for logics entails that the connectives of one logic can be defined in the other without reinterpreting propositional letters (Wojcicki, 1988, p. 70), this establishes the failure of intertranslatability. A fortiori, no logically weaker sublogic $L_0$ of $\textbf{K3}$ can be intertranslatable with classical propositional logic.

Here is our proof that $\textbf{K3}$ is not intertranslatable with classical propositional logic, following the technique in (Wojcicki, 1988, Thm. 1.8.9). If it were, then it would feature formulas $N(\cdot )$ and $O(\cdot ,\cdot )$ defining in $\textbf{K3}$ classical negation and disjunction. However, in $\textbf{K3}$, one can prove by induction on its complexity that for any formula $\varphi$ containing only one propositional letter *p*, $\varphi$ and *N*(*p*) are $\textbf{K3}$-logically equivalent, where *N*(*p*) can be one of:

$$\begin{aligned} p,\lnot p, p\vee \lnot p,\lnot (p\vee \lnot p). \end{aligned}$$

By employing the explosion law for *p* and $\lnot p$, and excluded middle for $p\vee \lnot p$ and $\lnot (p\vee \lnot p)$, one can see that none of these alternatives are possible.^17^

There are also logics that despite having the same logical strength have different scientific strength. One notable example is the case of classical propositional logic and intuitionistic propositional logic. We saw in the previous section that these have the same logical strength. However, classical propositional logic has greater scientific strength than intuitionistic propositional logic. For, on the one hand, classical propositional logic is scientifically at least as strong as intuitionistic propositional logic since intuitionistic logic is intertranslatable via the identity translation with a sublogic of classical logic, namely intuitionistic logic. But, on the other hand, there is no pair of sound schematic translations witnessing the intertranslatability of classical propositional logic and intuitionistic propositional logic (Meadows 2023: Prop. 22), and clearly no logically weaker sublogic of intuitionistic logic can be intertranslatable with classical logic.

Since scientific strength is obtained by supplementing logical strength with further conditions, we have cases in which it is known that two logics have different scientific strength but it is not known whether they have the same logical strength. Consider again the modal logics $\textbf{K}$ and $\textbf{T}$. We have seen that they have equal logical strength. However, a result of Pellettier and Urquhart (2003: Th. 4.5) entails that $\textbf{T}$ is scientifically stronger than $\textbf{K}$ because $\textbf{T}$ is not intertranslatable with $\textbf{K}$, and therefore with any logically weaker sublogic of $\textbf{K}$. The reason for the failure of intertranslatability of $\textbf{T}$ and $\textbf{K}$ is that, since both logics have the finite model property, translational equivalence requires isomorphism of classes of finite models. However, since $\textbf{K}$ is a sublogic of $\textbf{T}$, there are models of $\textbf{K}$ of size *n* that are not models of $\textbf{T}$. The same result entails that the logics $\textbf{T}, \textbf{B}, \textbf{S4},\textbf{S5}$, all differ in scientific strength. There are nonetheless logics that have equal scientific strength. An example involves some variations of classical logics that appear to be notational variants of each other. For instance, classical propositional logic with logical constants $\lnot$ and $\vee$ and the implicational fragment of classical propositional logic with a falsum constant, which we saw in Sect. 2.2 to have the same logical strength, are intertranslatable. For a more surprising example, by a result of Lenzen (1979), the modal logics $\mathbf{S4.4}$ and $\textbf{KD45}$ are intertranslatable, and therefore have equal scientific strength.

What has been said so far also enables us to compare theories in different logics by means of scientific strength, although this leads us into uncharted territory: not much is known about notions of theoretical equivalence such as intertranslatability for nonclassical mathematical theories. Nonetheless, the available results for classical give us a template for how the comparison among theories in different logics proceeds. For instance, as we saw above, the result of closing $\textsf{HA}^\textsf{id}$ under classical logic is simply $\textsf{PA}$. Therefore, by Visser’s result that $\textsf{PA}$ is not intertranslatable with any of its proper extensions, $\textsf{HA}$ cannot be as scientifically strong as any of these extensions of $\textsf{PA}$. In particular, this holds for the example considered above of $\textsf{PA}+\lnot \textrm{Con}(\textsf{PA})$. For similar reasons, $\textsf{HA}$ cannot be as scientifically strong as any theory that is properly logically stronger than $\mathsf {ZF_{Fin}}$ plus the assertion that every set has a transitive closure. The study of theoretical equivalence for intuitionistic theories is needed to obtain a converse for these examples. For instance, to conclude that $\textsf{HA}$ is scientifically weaker than $\textsf{PA}+\lnot \textrm{Con}(\textsf{PA})$, one would require an analogue of Visser’s result for intuitionistic theories, to the effect that $\textsf{HA}$ cannot be intertranslatable with the intuitionistic translation of $\textsf{PA}+\textrm{Con}(\textsf{PA})$.

An interesting case is provided the comparison of $\textsf{IZF}$ plus the axiom of choice and $\textsf{ZFC}$.^18^ It is well known that the two axiomatizations have the same theorems, since, in the context of $\textsf{IZF}$, the axiom of choice allows us to derive all instances of the law of excluded middle in the language of set theory. Yet, according to our definition of scientific strength, $\textsf{ZFC}$ is stronger purely in virtue of the difference in scientific strength occurring at the level of background logics. This highlights a feature of our approach to the comparison of theories that, in order to reflect the distinction between the comparison of logics (via schematic translation) and non-logical assumptions (via interpretations), distinguishes sharply between logical and non-logical components of theories: in comparing non-logical content, one needs first to fix suitable schematic translations dealing with logical information. It then follows that no amount of information at the level of non-logical axioms can make up for an asymmetry in scientific strength at the level of logics. However, as we will see in the next section, it may be possible to modify our approach so that axiomatizations in different logics but with the same theorems have equal scientific strength.

## Abductivism and Its Strengths

We have presented a framework to analyze the notions of logical and scientific strength. Our framework has a number of essential features. One essential feature is that, by employing translations between theories, the framework allows one to compare the logical and scientific strength of theories in a formally precise way. The framework is directly applicable to the debate on logical and mathematical abductivism. Williamson (2017) and Russell (2018) analyzed logical strength essentially in terms of the subtheory relation. This fails to capture many interesting cases of theory comparison. Our framework allows theory comparison between theories that are not cast in the same language. Nonetheless, it also clarifies how the subtheory relation fits into a more general account of logical strength. In particular, being a proper subtheory of another theory implies being logically not stronger than it.

Other essential features of our framework concern the relation between logical and scientific strength. Logical strength has to do with the deductive power of a theory, while scientific strength has to do with its information. By implementing these ideas via suitable translations, we see that scientific strength entails logical strength rather than the other way around. According to Williamson, logical strength entails scientific strength, essentially because more deductive power yields more information. If this is perhaps a plausible picture when comparing theories cast in the same language, it becomes harder to defend when one must translate between theories. For, if not suitably regimented, translations may compromise the information contained in theorems, and this is not compatible with theories having the same scientific strength. For instance, facts such as the interpretation of $\textsf{PA}$+‘$\textsf{PA}$ is inconsistent’ in $\textsf{PA}$ rely essentially on distorting the information contained in ‘$\textsf{PA}$ is inconsistent’. It then follows that logical strength cannot entail scientific strength.

By ensuring that the consequences of a theory are translated in accordance to suitable information-preserving constraints, our proposal maintains the generality given by understanding logical strength in terms of translations, while providing a notion of scientific strength as a refinement of the logical one. As a result, scientific strength implies logical strength but not vice versa: not all translations involved in the relation of logical strength are adequate for scientific strength. For instance, for $\textsf{PA}$ to be scientifically as strong as $\textsf{PA}$+‘$\textsf{PA}$ is inconsistent’, the structural role played by ‘$\textsf{PA}$ is inconsistent’ in derivations should be preserved, and $\textsf{PA}$ has to be inconsistent after all. Hence, our notion of scientific strength gives its due to the intuitive idea that scientific strength has to do with the information contained in a theory.

Our framework combines notions of reducibility and equivalence that are usually employed in different domains. Interpretability strength is the standard tool to compare mathematical theories, schematic translations are generally employed to compare pure logics, and intertranslatability is a standard measure of theoretical equivalence for scientific theories. Therefore, our framework paves the way to a unified approach to the comparison of formal theories. The specific combination of notions of reducibility employed in our characterization of logical and scientific strength delivers several very intuitive verdicts when applied to canonical examples. Admittedly, some other verdicts are more controversial, for instance that intuitionistic logic is logically as strong as and scientifically weaker than classical logic, and that extensionally equivalent theories cast in different logics, such as $\textsf{IZF}$ plus choice and $\textsf{ZFC}$, may differ in scientific strength.

However, our framework is flexible enough to accommodate changes in the notions of reduction employed in the characterization of logical and scientific strength. The essential features of our approach are compatible with variations of the implementation details, provided that the basic framework involving translation between theories is preserved, and that the notions of theoretical reduction employed in defining scientific strength amounts to sufficiently strict refinements of the ones employed in the characterization of logical strength. For instance, faithful interpretability—in which not only provability, but also unprovability is preserved via the translation—may replace the looser notion of interpretability. Analogously, instead of focusing on sound translations in the comparison of pure logics, one can consider the stricter notion of exact translation.

A more fine-grained characterization of scientific strength would be obtained by disentangling the two directions of intertranslatability. Following the terminology in Visser (2006), if one theory *T* can see that the composition two interpretations between *T* itself and *W* is equivalent to the identity interpretation, *T* is called a *retract* of *W*. Now, classical logic and intuitionistic logic fail to be intertranslatable because classical logic is not a retract of intuitionistic logic. One could modify the characterization of scientific strength by requiring, essentially, for $T_1$ to be as scientifically strong as $T_2$, that $T_1$ is a retract of $T_2$. This characterization would still allow intuitionistic logic to be as scientifically strong as classical logic: if intuitionistic logic turned out to be a retract of classical logic, then the two logics would have the same scientific strength, and there may be scope for $\textsf{IZF}$ plus choice and $\textsf{ZFC}$ to be of equal scientific strength.

Finally, instead of intertranslatability, which is occasionally considered to be too strict for theoretical equivalence (Weatherall, 2019), can be replaced by looser notions such as bi-interpretability (a.k.a. weak intertranslatability, homotopy equivalence) or categorical equivalence (Halvorson, 2019). All these alternatives will be considered in future, more technical work.

## Footnotes

1 In the current context it does not matter whether we characterize Cn in terms of logical consequence or derivability. Clearly, this matters when one considers logics that are not complete.

2 For instance, $\textsf{ZFC}$+‘there is a measurable cardinal’ and $\mathsf {ZFC}$ plus the Axiom of Constructibility.

3 For the relevant facts concerning interpretations of inconsistency in reasonable theories containing a modicum of arithmetic, we refer to (Lindström, 2003, Ch 7), and in particular (Lindström, 2003, Thm. 8).

4 We take a *theory* to be a set of axioms closed under a given logic.

5 The interpretation of finite set theory in arithmetic is due to Ackermann (1937). For the interpretation of the axiom of choice, the classical references are Gödel (1948) and Cohen (1963).

6 In particular, in $\textsf{PA}_\textsf{k3}(P)$ *P* can appear in induction, and the induction principle of $\textsf{PA}$ needs to be formulated as a rule to preserve soundness (Halbach & Nicolai, 2018).

7 For discussion of the requirements on translations between logics, see Dewar (2018) and Woods (2018).

8 A translation is compositional if, roughly, it commutes uniformly with logical vocabulary.

9 The example is taken from Pellettier and Urquhart (2003), Example 2.5.

10 If one allows non-effective translations, one obtains the unsound result that classic first-order logic and classical propositional logic have the same logical strength (Kocurec, 2017).

11 A sound translation of $\textbf{S4}$ into intuitionistic logic can be defined as follows. One can employ the ‘erasure’ translation schema to translate $\textbf{S4}$ in classical logic, and then employ the Gödel–Gentzen translation. Transitivity of sound translations then gives us the claim.

12 $\textsf{IZF}$ is obtained by taking the background logic to be intuitionistic and replacing $\textsf{ZF}$’s Axioms of Foundation and Replacement with the Axioms of $\in$-induction and Collection.

13 This point requires extra care in defining a suitable notion of interpretation for intuitionistic theories. Our claim is true for reasonable notions of interpretation for intuitionistic theories (Visser, 1999).

14 For an excellent overview of various notions of translations between logics extending sound and schematic translations, including original contributions, we refer to French (2010). Our notion of intertranslatability is a natural generalization of Pellettier and Urquhart’s definition taking into account logics which may lack a sufficiently powerful conditional. Our notion also differs from French’s, who employs the term ‘intertranslatability’ to refer to mutual faithful exact translatability. Following Caleiro and Gonçalves (2007), French calls our notion *equipollence*.

15 Enayat shows that, for extensions of $\textsf{ZF}$ in the language $\mathscr {L}_\in$ of set theory, the relation of bi-interpretability—a slight weakening of the notion of intertranslatability—reduces to the subtheory relation. This yields that the two theories cannot be bi-interpretable, and therefore not intertranslatable.

16 This also follows from results in Enayat (2016).

17 In more detail: since $p,\lnot p$ classically entails *q*, we would have

$$\begin{aligned} p,N(p)\vDash _{\textbf{K3}} q \end{aligned}$$

However, this cannot be the case if $N(p)\equiv p$, if $N(p)\equiv \lnot (p\wedge \lnot p)$, if $N(p)\equiv p\vee \lnot p$. If $N(p)\equiv \lnot p$, we can use *O*(*p*, *q*). In $\textbf{K3}$, there are only the following forms *O*(*p*, *q*) can take:

$$\begin{aligned} p\vee q,p\vee \lnot q,\lnot p\vee q,\lnot p\vee \lnot q,\lnot (p\vee q),\lnot (\lnot p\vee q)\vee \lnot (\lnot q\vee p) \end{aligned}$$

But $\textbf{K3}$ does not entail:

$$\begin{aligned} p\vee \lnot p,p\vee \lnot \lnot p,\lnot p\vee \lnot p,\lnot p\vee p,\lnot (p\vee \lnot p), \lnot (\lnot p \vee \lnot p)\vee \lnot (p\vee p), \end{aligned}$$

since

$$\neg (\neg p \vee \neg p)\vee \neg (p\vee p) \,{\reflectbox{$\vDash$}} \vDash_{\mathbf{K3}} \neg \neg p\vee \neg p {\reflectbox{$\vDash$}} \vDash_{\mathbf{K3}} p\vee \neg p$$

As noticed by an anonymous referee, if $\textbf{K3}$ is formulated with no truth constants for classical values, a much simpler proof of the results above can be given: there could be no sound translation of classical logic in $\textbf{K3}$ because the latter has no theorems. The proof we have given illustrates a method applicable in a wider range of cases.

18 We thank an anonymous referee for asking us to consider this case.
